# Supplementary material for: Non-Parametrical Canonical Analysis of Quality-Related Characteristics of Eggs of Different Varieties of Native Hens Compared to Laying Lineage
Source: Animals (Basel). 2019 Apr 9;9(4):153. doi: 10.3390/ani9040153 (PMC6523069; doi:10.3390/ani9040153)
Supplement: Supplementary file 1 [file animals-09-00153-s001.zip › Supplementary Table S10.docx]

**Supplementary Table S10.** Correlations between the variables and related canonical variables (canonical loadings) and between the variables and the other set of canonical variables (canonical cross loadings) for internal and external egg quality-related traits including yolk and white pH in Utrerana hens compared to laying lineage (n=97).

| Variable | U_1_ | U_2_ | U_3_ | U_4_ | U_5_ | U_6_ | V_1_ | V_2_ | V_3_ | V_4_ | V_5_ | V_6_ |
| --- | --- | --- | --- | --- | --- | --- | --- | --- | --- | --- | --- | --- |
| Egg weight | **-0.991** | **0.007** | **0.042** | **-0.104** | **0.065** | **0.032** | -0.955 | 0.005 | 0.023 | -0.046 | 0.018 | 0.006 |
| Major diameter | **-0.808** | **-0.052** | **0.342** | **-0.385** | **-0.176** | **-0.220** | -0.779 | -0.036 | 0.185 | -0.169 | -0.048 | -0.039 |
| Minor diameter | **-0.800** | **-0.092** | **-0.303** | **0.256** | **0.214** | **-0.385** | -0.771 | -0.064 | -0.164 | 0.113 | 0.058 | -0.068 |
| Shell^L*^ | **-0.130** | **0.905** | **0.247** | **0.038** | **0.318** | **0.013** | -0.125 | 0.625 | 0.133 | 0.017 | 0.086 | 0.002 |
| Shell^a*^ | **0.020** | **-0.320** | **0.051** | **0.687** | **-0.640** | **-0.110** | 0.019 | -0.221 | 0.028 | 0.302 | -0.173 | -0.020 |
| Shell^b*^ | **0.055** | **-0.947** | **0.063** | **0.251** | **-0.182** | **-0.028** | 0.053 | -0.654 | 0.034 | 0.110 | -0.049 | -0.005 |
| White height | -0.318 | 0.188 | 0.150 | 0.294 | 0.528 | -0.143 | **-0.307** | **0.130** | **0.081** | **0.129** | **0.142** | **-0.025** |
| Yolk colour | -0.104 | -0.428 | -0.044 | 0.224 | 0.323 | -0.344 | **-0.100** | **-0.296** | **-0.024** | **0.098** | **0.087** | **-0.061** |
| Yolk^L*^ | -0.131 | 0.733 | 0.315 | -0.356 | -0.029 | 0.032 | **-0.126** | **0.506** | **0.170** | **-0.156** | **-0.008** | **0.006** |
| Yolk^a*^ | 0.092 | -0.397 | 0.051 | 0.438 | -0.051 | 0.020 | **0.088** | **-0.274** | **0.028** | **0.193** | **-0.014** | **0.004** |
| Yolk^b*^ | 0.270 | -0.461 | -0.029 | 0.234 | -0.366 | 0.462 | **0.261** | **-0.318** | **-0.016** | **0.103** | **-0.099** | **0.082** |
| Yolk diameter | -0.316 | -0.087 | 0.085 | 0.174 | -0.191 | 0.403 | **-0.304** | **-0.060** | **0.046** | **0.076** | **-0.052** | **0.071** |
| Shell weight | -0.604 | 0.549 | -0.395 | 0.320 | -0.172 | -0.102 | **-0.582** | **0.379** | **-0.213** | **0.140** | **-0.046** | **-0.018** |
| Yolk weight | -0.474 | -0.341 | 0.502 | 0.210 | -0.328 | -0.002 | **-0.457** | **-0.236** | **0.271** | **0.092** | **-0.088** | **0.000** |
| White weight | -0.889 | -0.027 | -0.152 | -0.210 | 0.221 | -0.031 | **-0.857** | **-0.018** | **-0.082** | **-0.092** | **0.059** | **-0.006** |
| Yolk pH | 0.105 | -0.085 | -0.014 | -0.158 | -0.537 | -0.061 | **0.101** | **-0.059** | **-0.008** | **-0.070** | **-0.145** | **-0.011** |
| White pH | 0.109 | 0.044 | 0.284 | 0.115 | -0.410 | -0.557 | **0.105** | **0.031** | **0.153** | **0.051** | **-0.111** | **-0.098** |
| U_1_, U_2_, U_3_, U_4_, U_5_, U_6_: canonical variate containing external quality related traits; V_1_, V_2_, V_3_, V_4_, V_5_, V_6_: canonical variate containing internal quality related traits. | | | | | | | | | | | | |
