# Supplementary material for: Non-Parametrical Canonical Analysis of Quality-Related Characteristics of Eggs of Different Varieties of Native Hens Compared to Laying Lineage
Source: Animals (Basel). 2019 Apr 9;9(4):153. doi: 10.3390/ani9040153 (PMC6523069; doi:10.3390/ani9040153)
Supplement: Supplementary file 1 [file animals-09-00153-s001.zip › Supplementary Table S11.docx]

**Supplementary Table S11.** Proportion of Variance Eigenvalues and percentages of explained common variance associated with each factor of internal and external egg quality related traits including yolk and white pH in Utrerana hens compared to laying lineage (n=97).

| Canonical Variable | 1st | 2nd | 3rd | 4th | 5th | 6th |
| --- | --- | --- | --- | --- | --- | --- |
| Eigenvalue | 13.109 | 0.912 | 0.411 | 0.239 | 0.078 | 0.032 |
| Wilk’s Λ Statistic | 0.019 | 0.269 | 0.514 | 0.725 | 0.898 | 0.969 |
| Proportion of variance of external quality related variables explained by its own canonical variate ($U_{e}^{2}$) | 0.382 | 0.305 | 0.046 | 0.127 | 0.104 | 0.035 |
| Proportion of variance of external quality related variables explained by opposite canonical variate ($V_{e}^{2}$) | 0.355 | 0.145 | 0.014 | 0.025 | 0.008 | 0.001 |
| Proportion of variance of internal quality related variables explained by its own canonical variate ($U_{i}^{2}$) | 0.156 | 0.142 | 0.059 | 0.07 | 0.109 | 0.077 |
| Proportion of variance of external quality related variables explained by opposite canonical variate ($V_{i}^{2}$) | 0.145 | 0.068 | 0.017 | 0.013 | 0.008 | 0.002 |
