# Supplementary material for: Non-Parametrical Canonical Analysis of Quality-Related Characteristics of Eggs of Different Varieties of Native Hens Compared to Laying Lineage
Source: Animals (Basel). 2019 Apr 9;9(4):153. doi: 10.3390/ani9040153 (PMC6523069; doi:10.3390/ani9040153)
Supplement: Supplementary file 1 [file animals-09-00153-s001.zip › Supplementary Table S3.docx]

**Supplementary Table S3.** Summary of the results for the Kruskal-Wallis H test and the determinative coefficient through r or partial eta squared (ηp²), for fixed effects for internal and external egg quality traits from the model including yolk and white pH in Utrerana hens (n=97).

| Variable | Parameter | Egg weight | Major diameter | Minor diameter | Shell^L*^ | Shell^a*^ | Shell^b*^ | White height | Yolk colour | Yolk^L*^ | Yolk^a*^ | Yolk^b*^ | Yolk diameter | Shell weight | Yolk weight | White weight | Yolk pH | White pH |
| --- | --- | --- | --- | --- | --- | --- | --- | --- | --- | --- | --- | --- | --- | --- | --- | --- | --- | --- |
| Month | χ^2^ | 1.413 | 0.305 | 0.644 | 2.816 | 2.665 | 3.203 | 3.526 | 9.998 | 0.263 | 5.778 | 11.6 | 2.508 | 1.321 | 0.534 | 0.974 | 12.882 | 1.413 |
|  | dfn | 2 | 2 | 2 | 2 | 2 | 2 | 2 | 2 | 2 | 2 | 2 | 2 | 2 | 2 | 2 | 2 | 2 |
|  | p-value | 0.493 | 0.859 | 0.725 | 0.245 | 0.264 | 0.202 | 0.171 | 0.007 | 0.877 | 0.056 | 0.003 | 0.285 | 0.517 | 0.766 | 0.614 | 0.002 | 0.493 |
|  | dfd | 96 | 96 | 96 | 96 | 96 | 96 | 96 | 96 | 96 | 96 | 96 | 96 | 96 | 96 | 96 | 96 | 96 |
|  | F | 0.707 | 0.153 | 0.322 | 1.408 | 1.333 | 1.602 | 1.763 | 4.999 | 0.132 | 2.889 | 5.800 | 1.254 | 0.661 | 0.267 | 0.487 | 6.441 | 11.635 |
|  | ηp² | 0.015 | 0.003 | 0.007 | 0.028 | 0.027 | 0.032 | 0.035 | 0.094 | 0.003 | 0.057 | 0.108 | 0.025 | 0.014 | 0.006 | 0.010 | 0.118 | 0.195 |
| Order | χ^2^ | 12.416 | 8.64 | 8.558 | 6.98 | 5.076 | 9.589 | 6.729 | 4.987 | 3.778 | 4 | 4.756 | 4.83 | 2.961 | 7.12 | 15.284 | 4.684 | 12.416 |
|  | df | 5 | 5 | 5 | 5 | 5 | 5 | 5 | 5 | 5 | 5 | 5 | 5 | 5 | 5 | 5 | 5 | 5 |
|  | p-value | 0.03 | 0.124 | 0.128 | 0.222 | 0.407 | 0.088 | 0.242 | 0.417 | 0.582 | 0.549 | 0.446 | 0.437 | 0.706 | 0.212 | 0.009 | 0.456 | 0.03 |
|  | dfd | 93 | 93 | 93 | 93 | 93 | 93 | 93 | 93 | 93 | 93 | 93 | 93 | 93 | 93 | 93 | 93 | 93 |
|  | F | 2.483 | 1.728 | 1.712 | 1.396 | 1.015 | 1.918 | 1.346 | 0.997 | 0.756 | 0.800 | 0.951 | 0.966 | 0.592 | 1.424 | 3.057 | 0.937 | 3.239 |
|  | ηp² | 0.118 | 0.085 | 0.084 | 0.070 | 0.052 | 0.093 | 0.067 | 0.051 | 0.039 | 0.041 | 0.049 | 0.049 | 0.031 | 0.071 | 0.141 | 0.048 | 0.148 |
| Period | χ^2^ | 1.107 | 0.569 | 1.026 | 4.941 | 0.715 | 4.407 | 1.391 | 0.038 | 4.367 | 1.621 | 10.949 | 1.122 | 0.961 | 2.566 | 0.678 | 10.911 | 1.107 |
|  | df | 2 | 2 | 2 | 2 | 2 | 2 | 2 | 2 | 2 | 2 | 2 | 2 | 2 | 2 | 2 | 2 | 2 |
|  | p-value | 0.575 | 0.753 | 0.599 | 0.085 | 0.699 | 0.11 | 0.499 | 0.981 | 0.113 | 0.445 | 0.004 | 0.571 | 0.618 | 0.277 | 0.712 | 0.004 | 0.575 |
|  | dfd | 96 | 96 | 96 | 96 | 96 | 96 | 96 | 96 | 96 | 96 | 96 | 96 | 96 | 96 | 96 | 96 | 96 |
|  | F | 0.554 | 0.285 | 0.513 | 2.471 | 0.358 | 2.204 | 0.696 | 0.019 | 2.184 | 0.811 | 5.475 | 0.561 | 0.481 | 1.283 | 0.339 | 5.456 | 10.668 |
|  | ηp² | 0.011 | 0.006 | 0.011 | 0.049 | 0.007 | 0.044 | 0.014 | 0.000 | 0.044 | 0.017 | 0.102 | 0.012 | 0.010 | 0.026 | 0.007 | 0.102 | 0.182 |
| Laying Age | χ^2^ | 0.673 | 1.43 | 1.063 | 0.363 | 0.324 | 0.908 | 0.195 | 0.44 | 0.008 | 1.531 | 0.034 | 0.126 | 1.932 | 0.627 | 0.808 | 5.438 | 0.673 |
|  | df | 1 | 1 | 1 | 1 | 1 | 1 | 1 | 1 | 1 | 1 | 1 | 1 | 1 | 1 | 1 | 1 | 1 |
|  | p-value | 0.412 | 0.232 | 0.303 | 0.547 | 0.569 | 0.341 | 0.659 | 0.507 | 0.931 | 0.216 | 0.853 | 0.723 | 0.165 | 0.428 | 0.369 | 0.020 | 0.412 |
|  | dfd | 97 | 97 | 97 | 97 | 97 | 97 | 97 | 97 | 97 | 97 | 97 | 97 | 97 | 97 | 97 | 97 | 97 |
|  | F | 0.673 | 1.430 | 1.063 | 0.363 | 0.324 | 0.908 | 0.195 | 0.440 | 0.008 | 1.531 | 0.034 | 0.126 | 1.932 | 0.627 | 0.808 | 5.438 | 0.718 |
|  | r | 0.007 | 0.015 | 0.011 | 0.004 | 0.003 | 0.009 | 0.002 | 0.005 | 0.000 | 0.016 | 0.000 | 0.001 | 0.020 | 0.006 | 0.008 | 0.053 | 0.007 |
| Variety | χ^2^ | 19.754 | 21.254 | 11.194 | 28.807 | 34.087 | 42.35 | 5.268 | 10.528 | 9.626 | 11.3 | 10.529 | 16.291 | 32.163 | 27.74 | 32.529 | 8.695 | 19.754 |
|  | df | 4 | 4 | 4 | 4 | 4 | 4 | 4 | 4 | 4 | 4 | 4 | 4 | 4 | 4 | 4 | 4 | 4 |
|  | p-value | 0.001 | 0.000 | 0.024 | 0.000 | 0.000 | 0.000 | 0.261 | 0.032 | 0.047 | 0.023 | 0.032 | 0.003 | 0.000 | 0.000 | 0.000 | 0.069 | 0.001 |
|  | dfd | 94 | 94 | 94 | 94 | 94 | 94 | 94 | 94 | 94 | 94 | 94 | 94 | 94 | 94 | 94 | 94 | 94 |
|  | F | 4.939 | 5.314 | 2.799 | 7.202 | 8.522 | 10.588 | 1.317 | 2.632 | 2.407 | 2.825 | 2.632 | 4.073 | 8.041 | 6.935 | 8.132 | 2.174 | 2.761 |
|  | ηp² | 0.174 | 0.184 | 0.106 | 0.235 | 0.266 | 0.311 | 0.053 | 0.101 | 0.093 | 0.107 | 0.101 | 0.148 | 0.255 | 0.228 | 0.257 | 0.085 | 0.105 |
| Breed | χ^2^ | 9.834 | 4.72 | 7.561 | 26.658 | 23.037 | 37.926 | 1.271 | 5.086 | 4.127 | 6.249 | 6.501 | 1.69 | 28.423 | 6.524 | 12.567 | 0 | 9.834 |
|  | df | 1 | 1 | 1 | 1 | 1 | 1 | 1 | 1 | 1 | 1 | 1 | 1 | 1 | 1 | 1 | 1 | 1 |
|  | p-value | 0.002 | 0.030 | 0.006 | 0.000 | 0.000 | 0.000 | 0.260 | 0.024 | 0.042 | 0.012 | 0.011 | 0.194 | 0.000 | 0.011 | 0.000 | 1.000 | 0.002 |
|  | dfd | 97 | 97 | 97 | 97 | 97 | 97 | 97 | 97 | 97 | 97 | 97 | 97 | 97 | 97 | 97 | 97 | 97 |
|  | F | 9.834 | 4.720 | 7.561 | 26.658 | 23.037 | 37.926 | 1.271 | 5.086 | 4.127 | 6.249 | 6.501 | 1.690 | 28.423 | 6.524 | 12.567 | 0.000 | 7.318 |
|  | r | 0.092 | 0.046 | 0.072 | 0.216 | 0.192 | 0.281 | 0.013 | 0.050 | 0.041 | 0.061 | 0.063 | 0.017 | 0.227 | 0.063 | 0.115 | 0.000 | 0.070 |
| ηp² can be benchmarked against Cohen J. (1969) criteria of small (0.01), medium (0.09), and large (0.25) effects as suggested in Richardson (2011).In Cohen's terminology, a small effect size is one in which there is a real effect but which you can only see through careful study. By contrast, a 'large' effect size is an effect which is big enough, and/or consistent enough, that you may be able to see it 'with the naked eye'. | | | | | | | | | | | | | | | | | | |
