# Supplementary material for: Non-Parametrical Canonical Analysis of Quality-Related Characteristics of Eggs of Different Varieties of Native Hens Compared to Laying Lineage
Source: Animals (Basel). 2019 Apr 9;9(4):153. doi: 10.3390/ani9040153 (PMC6523069; doi:10.3390/ani9040153)
Supplement: Supplementary file 1 [file animals-09-00153-s001.zip › Supplementary Table S4.docx]

**Supplementary Table S4.** Summary of the results of the independent sample median test of the factors month, order, period, essay, variety and breed on internal and external egg quality-related traits excluding yolk and white pH in Utrerana hens compared to laying lineage (n=194).

| Variable | | Egg weight | | Major diameter | | Minor diameter | | Shell^L*^ | | Shell^a*^ | | Shell^b*^ | | White height | | Yolk colour | | Yolk^L*^ | | Yolk^a*^ | | Yolk^b*^ | | Yolk diameter | | Shell weight | | Yolk weight | | White weight | |
| --- | --- | --- | --- | --- | --- | --- | --- | --- | --- | --- | --- | --- | --- | --- | --- | --- | --- | --- | --- | --- | --- | --- | --- | --- | --- | --- | --- | --- | --- | --- | --- |
| Median | | 64.715 | | 59.560 | | 44.385 | | 87.420 | | -0.280 | | 4.770 | | 7.290 | | 12.000 | | 56.640 | | 8.560 | | 20.890 | | 43.015 | | 64.715 | | 59.560 | | 44.385 | |
| Value respect to the median | | > | ≤ | > | ≤ | > | ≤ | > | ≤ | > | ≤ | > | ≤ | > | ≤ | > | ≤ | > | ≤ | > | ≤ | > | ≤ | > | ≤ | > | ≤ | > | ≤ | > | ≤ |
| Month | March | 16 | 11 | 19 | 8 | 13 | 14 | 8 | 19 | 14 | 13 | 16 | 11 | 16 | 11 | 14 | 13 | 0 | 27 | 26 | 1 | 26 | 1 | 13 | 14 | 16 | 11 | 13 | 14 | 16 | 11 |
|  | April | 39 | 48 | 40 | 47 | 44 | 43 | 50 | 37 | 48 | 39 | 39 | 48 | 48 | 39 | 15 | 72 | 48 | 39 | 34 | 53 | 23 | 64 | 44 | 43 | 48 | 39 | 41 | 46 | 43 | 44 |
|  | May | 29 | 33 | 27 | 35 | 31 | 31 | 25 | 36 | 27 | 34 | 26 | 35 | 28 | 34 | 3 | 59 | 41 | 21 | 18 | 44 | 41 | 21 | 31 | 31 | 26 | 36 | 30 | 32 | 27 | 35 |
|  | June | 13 | 5 | 11 | 7 | 9 | 9 | 13 | 5 | 7 | 11 | 15 | 3 | 4 | 14 | 14 | 4 | 7 | 11 | 18 | 0 | 6 | 12 | 9 | 9 | 7 | 11 | 13 | 5 | 11 | 7 |
| Order | 1st laying | 24 | 43 | 25 | 42 | 28 | 39 | 31 | 36 | 35 | 32 | 30 | 37 | 27 | 40 | 12 | 55 | 33 | 34 | 35 | 32 | 38 | 29 | 25 | 42 | 29 | 38 | 29 | 38 | 23 | 44 |
|  | 2nd laying | 29 | 25 | 32 | 22 | 25 | 29 | 29 | 25 | 27 | 27 | 28 | 26 | 28 | 26 | 11 | 43 | 26 | 28 | 28 | 26 | 26 | 28 | 30 | 24 | 27 | 27 | 27 | 27 | 27 | 27 |
|  | 3rd laying | 25 | 16 | 22 | 19 | 24 | 17 | 16 | 24 | 21 | 19 | 22 | 18 | 19 | 22 | 11 | 30 | 22 | 19 | 20 | 21 | 20 | 21 | 21 | 20 | 20 | 21 | 24 | 17 | 25 | 16 |
|  | 4th laying | 13 | 8 | 12 | 9 | 14 | 7 | 13 | 8 | 10 | 11 | 11 | 10 | 14 | 7 | 8 | 13 | 10 | 11 | 8 | 13 | 9 | 12 | 15 | 6 | 15 | 6 | 10 | 11 | 16 | 5 |
|  | 5th laying | 5 | 4 | 5 | 4 | 5 | 4 | 7 | 2 | 3 | 6 | 4 | 5 | 6 | 3 | 3 | 6 | 4 | 5 | 5 | 4 | 2 | 7 | 5 | 4 | 6 | 3 | 6 | 3 | 5 | 4 |
|  | 6th laying | 1 | 1 | 1 | 1 | 1 | 1 | 0 | 2 | 0 | 2 | 1 | 1 | 2 | 0 | 1 | 1 | 1 | 1 | 0 | 2 | 1 | 1 | 1 | 1 | 0 | 2 | 1 | 1 | 1 | 1 |
| Period | 1 | 38 | 34 | 41 | 31 | 38 | 34 | 34 | 38 | 41 | 31 | 36 | 36 | 44 | 28 | 19 | 53 | 27 | 45 | 42 | 30 | 38 | 34 | 35 | 37 | 41 | 31 | 37 | 35 | 40 | 32 |
|  | 2 | 30 | 39 | 29 | 40 | 33 | 36 | 35 | 33 | 31 | 37 | 30 | 38 | 33 | 36 | 10 | 59 | 41 | 28 | 26 | 43 | 27 | 42 | 34 | 35 | 38 | 31 | 29 | 40 | 30 | 39 |
|  | 3 | 29 | 24 | 27 | 26 | 26 | 27 | 27 | 26 | 24 | 29 | 30 | 23 | 19 | 34 | 17 | 36 | 28 | 25 | 28 | 25 | 31 | 22 | 28 | 25 | 18 | 35 | 31 | 22 | 27 | 26 |
| Laying Age | Laying hens | 83 | 67 | 78 | 72 | 81 | 69 | 69 | 80 | 74 | 75 | 74 | 75 | 76 | 74 | 39 | 111 | 69 | 81 | 77 | 73 | 80 | 70 | 76 | 74 | 85 | 65 | 77 | 73 | 79 | 71 |
|  | Laying pullets | 14 | 30 | 19 | 25 | 16 | 28 | 27 | 17 | 22 | 22 | 22 | 22 | 20 | 24 | 7 | 37 | 27 | 17 | 19 | 25 | 16 | 28 | 21 | 23 | 12 | 32 | 20 | 24 | 18 | 26 |
| Variety | Franciscan | 12 | 34 | 14 | 32 | 12 | 34 | 19 | 27 | 39 | 7 | 31 | 15 | 15 | 31 | 13 | 33 | 26 | 20 | 32 | 14 | 29 | 17 | 28 | 18 | 20 | 26 | 34 | 12 | 8 | 38 |
|  | White | 1 | 3 | 2 | 2 | 1 | 3 | 3 | 1 | 1 | 3 | 0 | 4 | 2 | 2 | 0 | 4 | 2 | 2 | 0 | 4 | 1 | 3 | 0 | 4 | 0 | 4 | 0 | 4 | 3 | 1 |
|  | Black | 21 | 26 | 14 | 33 | 28 | 19 | 19 | 28 | 24 | 23 | 25 | 22 | 24 | 23 | 18 | 29 | 15 | 32 | 23 | 24 | 17 | 30 | 17 | 30 | 20 | 27 | 19 | 28 | 23 | 24 |
|  | Patridge | 26 | 24 | 37 | 13 | 21 | 29 | 18 | 31 | 32 | 17 | 40 | 9 | 24 | 26 | 9 | 41 | 28 | 22 | 26 | 24 | 34 | 16 | 34 | 16 | 15 | 35 | 35 | 15 | 27 | 23 |
|  | Leghorn | 37 | 10 | 30 | 17 | 35 | 12 | 37 | 10 | 0 | 47 | 0 | 47 | 31 | 16 | 6 | 41 | 25 | 22 | 15 | 32 | 15 | 32 | 18 | 29 | 42 | 5 | 9 | 38 | 36 | 11 |
| Breed | Utrerana | 60 | 87 | 67 | 80 | 62 | 85 | 59 | 87 | 96 | 50 | 96 | 50 | 65 | 82 | 40 | 107 | 71 | 76 | 81 | 66 | 81 | 66 | 79 | 68 | 55 | 92 | 88 | 59 | 61 | 86 |
|  | Leghorn | 37 | 10 | 30 | 17 | 35 | 12 | 37 | 10 | 0 | 47 | 0 | 47 | 31 | 16 | 6 | 41 | 25 | 22 | 15 | 32 | 15 | 32 | 18 | 29 | 42 | 5 | 9 | 38 | 36 | 11 |
