# Supplementary material for: Non-Parametrical Canonical Analysis of Quality-Related Characteristics of Eggs of Different Varieties of Native Hens Compared to Laying Lineage
Source: Animals (Basel). 2019 Apr 9;9(4):153. doi: 10.3390/ani9040153 (PMC6523069; doi:10.3390/ani9040153)
Supplement: Supplementary file 1 [file animals-09-00153-s001.zip › Supplementary Table S5.docx]

**Supplementary Table S5.** Summary of the results of the independent sample median test of the factors month, order, period, essay, variety and breed on internal and external egg quality-related traits including yolk and white pH in Utrerana hens compared to laying lineage (n=97).

| Variable | | Egg weight | | Major diameter | | Minor diameter | | Shell^L*^ | | Shell^a*^ | | Shell^b*^ | | White height | | Yolk colour | | Yolk^L*^ | | Yolk^a*^ | | Yolk^b*^ | | Yolk diameter | | Shell weight | | Yolk weight | | White weight | | Yolk pH | | White pH | |
| --- | --- | --- | --- | --- | --- | --- | --- | --- | --- | --- | --- | --- | --- | --- | --- | --- | --- | --- | --- | --- | --- | --- | --- | --- | --- | --- | --- | --- | --- | --- | --- | --- | --- | --- | --- |
| Median | | 63.970 | | 59.350 | | 44.380 | | 88.220 | | -0.160 | | 4.690 | | 7.400 | | 12.000 | | 56.990 | | 7.920 | | 19.920 | | 42.850 | | 8.620 | | 19.740 | | 35.230 | | 6.000 | | 9.000 | |
| Value respect to the median | | > | ≤ | > | ≤ | > | ≤ | > | ≤ | > | ≤ | > | ≤ | > | ≤ | > | ≤ | > | ≤ | > | ≤ | > | ≤ | > | ≤ | > | ≤ | > | ≤ | > | ≤ | > | ≤ | > | ≤ |
| Month | April | 41 | 43 | 42 | 42 | 41 | 43 | 43 | 41 | 42 | 42 | 40 | 44 | 42 | 42 | 15 | 69 | 40 | 44 | 40 | 44 | 38 | 46 | 42 | 42 | 43 | 41 | 40 | 44 | 41 | 43 | 19 | 65 | 0 | 84 |
|  | May | 4 | 6 | 4 | 6 | 5 | 5 | 4 | 6 | 5 | 5 | 6 | 4 | 5 | 5 | 0 | 10 | 7 | 3 | 5 | 5 | 9 | 1 | 6 | 4 | 4 | 6 | 6 | 4 | 5 | 5 | 10 | 0 | 7 | 3 |
|  | June | 3 | 0 | 2 | 1 | 2 | 1 | 1 | 2 | 1 | 2 | 2 | 1 | 0 | 3 | 3 | 0 | 1 | 2 | 3 | 0 | 1 | 2 | 0 | 3 | 1 | 2 | 2 | 1 | 2 | 1 | 1 | 2 | 0 | 3 |
| Order | 1st laying | 12 | 22 | 11 | 23 | 13 | 21 | 19 | 15 | 14 | 20 | 12 | 22 | 12 | 22 | 3 | 31 | 19 | 15 | 18 | 16 | 18 | 16 | 13 | 21 | 15 | 19 | 15 | 19 | 11 | 23 | 11 | 23 | 2 | 32 |
|  | 2nd laying | 13 | 14 | 15 | 12 | 13 | 14 | 13 | 14 | 15 | 12 | 14 | 13 | 17 | 10 | 5 | 22 | 13 | 14 | 12 | 15 | 11 | 16 | 13 | 14 | 13 | 14 | 10 | 17 | 15 | 12 | 9 | 18 | 2 | 25 |
|  | 3rd laying | 13 | 6 | 13 | 6 | 13 | 6 | 6 | 13 | 12 | 7 | 13 | 6 | 7 | 12 | 4 | 15 | 10 | 9 | 12 | 7 | 10 | 9 | 11 | 8 | 10 | 9 | 13 | 6 | 12 | 7 | 6 | 13 | 3 | 16 |
|  | 4th laying | 8 | 5 | 7 | 6 | 7 | 6 | 7 | 6 | 6 | 7 | 7 | 6 | 8 | 5 | 4 | 9 | 5 | 8 | 5 | 8 | 8 | 5 | 9 | 4 | 8 | 5 | 8 | 5 | 8 | 5 | 2 | 11 | 0 | 13 |
|  | 5th laying | 1 | 2 | 1 | 2 | 1 | 2 | 3 | 0 | 1 | 2 | 1 | 2 | 2 | 1 | 1 | 2 | 1 | 2 | 1 | 2 | 1 | 2 | 1 | 2 | 2 | 1 | 1 | 2 | 1 | 2 | 1 | 2 | 0 | 3 |
|  | 6th laying | 1 | 0 | 1 | 0 | 1 | 0 | 0 | 1 | 0 | 1 | 1 | 0 | 1 | 0 | 1 | 0 | 0 | 1 | 0 | 1 | 0 | 1 | 1 | 0 | 0 | 1 | 1 | 0 | 1 | 0 | 1 | 0 | 0 | 1 |
| Period | 1 | 23 | 19 | 22 | 20 | 22 | 20 | 21 | 21 | 24 | 18 | 20 | 22 | 22 | 20 | 5 | 37 | 23 | 19 | 19 | 23 | 18 | 24 | 20 | 22 | 21 | 21 | 23 | 19 | 22 | 20 | 13 | 29 | 0 | 42 |
|  | 2 | 18 | 25 | 20 | 23 | 20 | 23 | 23 | 20 | 18 | 25 | 20 | 23 | 21 | 22 | 10 | 33 | 18 | 25 | 21 | 22 | 21 | 22 | 22 | 21 | 22 | 21 | 17 | 26 | 20 | 23 | 7 | 36 | 0 | 43 |
|  | 3 | 7 | 5 | 6 | 6 | 6 | 6 | 4 | 8 | 6 | 6 | 8 | 4 | 4 | 8 | 3 | 9 | 7 | 5 | 8 | 4 | 9 | 3 | 6 | 6 | 5 | 7 | 8 | 4 | 6 | 6 | 10 | 2 | 7 | 5 |
| Laying Age | Laying hens | 38 | 34 | 38 | 34 | 37 | 35 | 35 | 37 | 36 | 36 | 39 | 33 | 34 | 38 | 14 | 58 | 37 | 35 | 37 | 35 | 34 | 38 | 35 | 37 | 38 | 34 | 37 | 35 | 37 | 35 | 27 | 45 | 6 | 66 |
|  | Laying pullets | 10 | 15 | 10 | 15 | 11 | 14 | 13 | 12 | 12 | 13 | 9 | 16 | 13 | 12 | 4 | 21 | 11 | 14 | 11 | 14 | 14 | 11 | 13 | 12 | 10 | 15 | 11 | 14 | 11 | 14 | 3 | 22 | 1 | 24 |
| Variety | Franciscan | 7 | 21 | 7 | 21 | 8 | 20 | 12 | 16 | 24 | 4 | 18 | 10 | 9 | 19 | 7 | 21 | 15 | 13 | 18 | 10 | 19 | 9 | 17 | 11 | 12 | 16 | 20 | 8 | 4 | 24 | 8 | 20 | 2 | 26 |
|  | White | 1 | 2 | 1 | 2 | 1 | 2 | 2 | 1 | 0 | 3 | 0 | 3 | 2 | 1 | 0 | 3 | 2 | 1 | 0 | 3 | 2 | 1 | 0 | 3 | 0 | 3 | 0 | 3 | 2 | 1 | 1 | 2 | 0 | 3 |
|  | Black | 11 | 13 | 9 | 15 | 13 | 11 | 9 | 15 | 9 | 15 | 12 | 12 | 14 | 10 | 8 | 16 | 7 | 17 | 15 | 9 | 10 | 14 | 9 | 15 | 10 | 14 | 7 | 17 | 12 | 12 | 12 | 12 | 2 | 22 |
|  | Patridge | 14 | 9 | 19 | 4 | 13 | 10 | 8 | 15 | 15 | 8 | 18 | 5 | 12 | 11 | 1 | 22 | 11 | 12 | 10 | 13 | 12 | 11 | 16 | 7 | 9 | 14 | 16 | 7 | 16 | 7 | 3 | 20 | 2 | 21 |
|  | Leghorn | 15 | 4 | 12 | 7 | 13 | 6 | 17 | 2 | 0 | 19 | 0 | 19 | 10 | 9 | 2 | 17 | 13 | 6 | 5 | 14 | 5 | 14 | 6 | 13 | 17 | 2 | 5 | 14 | 14 | 5 | 6 | 13 | 1 | 18 |
| Breed | Utrerana | 33 | 45 | 36 | 42 | 35 | 43 | 31 | 47 | 48 | 30 | 48 | 30 | 37 | 41 | 16 | 62 | 35 | 43 | 43 | 35 | 43 | 35 | 42 | 36 | 31 | 47 | 43 | 35 | 34 | 44 | 24 | 54 | 6 | 72 |
|  | Leghorn | 15 | 4 | 12 | 7 | 13 | 6 | 17 | 2 | 0 | 19 | 0 | 19 | 10 | 9 | 2 | 17 | 13 | 6 | 5 | 14 | 5 | 14 | 6 | 13 | 17 | 2 | 5 | 14 | 14 | 5 | 6 | 13 | 1 | 18 |
