# Supplementary material for: Non-Parametrical Canonical Analysis of Quality-Related Characteristics of Eggs of Different Varieties of Native Hens Compared to Laying Lineage
Source: Animals (Basel). 2019 Apr 9;9(4):153. doi: 10.3390/ani9040153 (PMC6523069; doi:10.3390/ani9040153)
Supplement: Supplementary file 1 [file animals-09-00153-s001.zip › Supplementary Table S9.docx]

**Supplementary Table S9.** Standardized canonical coefficients of variables, canonical correlations between two sets of variables (r), squared canonical correlation (r^2^) and their probabilities (F) for internal and external egg quality-related traits including yolk and white pH in Utrerana hens compared to laying lineage (n=97).

| Canonical pairs | 1st | 2nd | 3rd | 4th | 5th | 6th |
| --- | --- | --- | --- | --- | --- | --- |
| r (*R_c_*) | 0.964 | 0.691 | 0.540 | 0.439 | 0.270 | 0.176 |
| r^2^ ($R_{c}^{2}$) | 0.929 | 0.477 | 0.292 | 0.193 | 0.073 | 0.031 |
| F | 7.202 | 2.490 | 1.670 | 1.179 | 0.661 | 0.456 |
| Degrees of Freedom | 66 | 50 | 36 | 24 | 14 | 6 |
| Sig. | 0.000 | 0.000 | 0.012 | 0.262 | 0.810 | 0.839 |
| Standardized canonical coefficients of External quality related traits | | | | | | |
| Egg weight | **-0.987** | -0.048 | -0.389 | 0.339 | -0.111 | 2.531 |
| Major diameter | 0.036 | -0.005 | **0.876** | -0.779 | -0.494 | -1.557 |
| Minor diameter | -0.068 | -0.024 | **-0.575** | 0.27 | 0.518 | -1.529 |
| Shell^L*^ | 0.041 | 0.167 | **1.775** | 1.025 | 1.192 | -0.179 |
| Shell^a*^ | -0.136 | 0.361 | -0.083 | 0.625 | -1.095 | 0.070 |
| Shell^b*^ | 0.083 | **-1.017** | 1.73 | 0.787 | 1.486 | 0.057 |
| Standardized canonical coefficients of Internal quality related traits | | | | | | |
| White height | 0.002 | 0.160 | 0.511 | 0.632 | 0.475 | 0.008 |
| Yolk colour | 0.053 | -0.468 | **-0.638** | 0.201 | 0.389 | -0.430 |
| Yolk^L*^ | -0.033 | **0.625** | 0.512 | -0.169 | 0.116 | 0.495 |
| Yolk^a*^ | -0.044 | 0.435 | **0.807** | 0.368 | -0.065 | 0.553 |
| Yolk^b*^ | -0.005 | -0.279 | -0.480 | 0.227 | -0.093 | 0.486 |
| Yolk diameter | -0.028 | -0.025 | 0.011 | 0.257 | -0.121 | 0.376 |
| Shell weight | -0.229 | 0.445 | **-0.740** | 0.691 | -0.332 | -0.129 |
| Yolk weight | -0.386 | -0.276 | **0.701** | 0.154 | -0.205 | -0.082 |
| White weight | **-0.755** | -0.292 | -0.107 | -0.780 | 0.001 | 0.073 |
| Yolk pH | 0.036 | -0.089 | 0.054 | -0.185 | -0.514 | -0.077 |
| White pH | 0.021 | -0.005 | 0.064 | 0.028 | -0.405 | -0.780 |
| Given the sample size of 97 a criterion of ≥\|0.53\| was considered for variable loadings to be significant ([Hair, Black, Babin, & Anderson, 2010](#_ENREF_1)). | | | | | | |
